# ACE2-fused nanobody targeting a cryptic RBD epitope broadly neutralizes SARS-like viruses

**DOI:** 10.1128/jvi.00608-26

**Published:** 2026-07-30

**Authors:** Weihong Zeng, Guanying Zhang, Huan Ma, Lijun Xiong, Mingshi Xu, Shanshan Wang, Xiaoxue Huang, Shi Chen, Xiangyang Chi, Sandra Chiu, Tengchuan Jin

**Affiliations:** 1 School of Biomedical and Environmental Engineering, Hefei Institute of Technology 834024 , Hefei, Anhui, China; 2 Laboratory of Structural Immunology, State Key Laboratory of Immune Response and Immunotherapy, Institute of Advanced Technology, University of Science and Technology of China 594412, Hefei, Anhui, China; 3 National Key Laboratory of Advanced Biotechnology, Academy of Military Medical Sciences 71040, Beijing, China; 4 Institute of Clinical Immunology, The First Affiliated Hospital of Anhui Medical University 36639, Hefei, Anhui, China; 5 Division of Life Sciences and Medicine, University of Science and Technology of China 12652, Hefei, Anhui, China; 6 Key Laboratory of Anhui Province for Emerging and Reemerging Infectious Diseases, Hefei, Anhui, China; 7 Department of Laboratory Medicine, The First Affiliated Hospital of USTC, Division of Life Sciences and Medicine, University of Science and Technology of China 12652, Hefei, Anhui, China; 8 Anhui Genebiol Biotech. Ltd., Hefei, Anhui, China; 9 Department of Infectious Diseases, The First Affiliated Hospital of USTC, Center for Advanced Interdisciplinary Science and Biomedicine of IHM, Division of Life Sciences and Medicine, University of Science and Technology of China 12652, Hefei, Anhui, China; 10 Clinical Research Hospital of Chinese Academy of Sciences (Hefei), University of Science and Technology of China 12652, Hefei, China; 11 Institute of Health and Medicine, Hefei Comprehensive National Science Center 604255 , Hefei, Anhui, China; St Jude Children's Research Hospital, Memphis, Tennessee, USA

**Keywords:** SARS-like coronaviruses, nanobodies, alternating immunization, conserved RBD epitope, ACE2 fusion protein

## Abstract

The SARS-CoV-2 (SARS2) and SARS-CoV (SARS1) viruses pose significant threats due to their high mutation rates and potential for cross-species transmission, which has rendered many antibodies ineffective. To counter potential future emergences of novel SARS-like viruses, it is imperative to develop nanobodies with broad-spectrum neutralizing capacity. Here, we employed an alternating immunization strategy in alpacas using SARS1 and SARS2 RBDs to generate cross-reactive nanobodies. Four crossing-binding nanobodies (aSR29, aSR196, aSR347, and aSR348) were identified, exhibiting high affinities for SARS1/SARS2 RBDs and Omicron subvariants. Structural analysis revealed that aSR29 targets a cryptic, highly conserved epitope at the inner base of the RBD, a region shielded from conventional antibodies by steric hindrance, efficiently neutralizing SARS1, SARS2, and multiple Variants of Concern (VOCs), including XBB.1.5 and BQ.1.1. Building on this unique binding mode, we engineered a bispecific fusion protein, aSR29-ACE2-Fc. This construct synergistically combines high-affinity RBD binding with the steric blockade of viral attachment, resulting in a 33-fold increase in neutralization potency against SARS2 and enhanced resilience against immune escape. Our findings validate an efficient strategy for eliciting broad-spectrum nanobodies and demonstrate the therapeutic potential of VHH-ACE2 fusion proteins against current and future SARS-like viruses.

**IMPORTANCE:**

The continued evolution of SARS-CoV-2 has rendered most existing antibodies ineffective, highlighting the urgent need for broad-spectrum countermeasures against current and future SARS-like viruses. Here, we developed an alternating immunization strategy to generate nanobodies that target a hidden, highly conserved region on the viral spike protein. One such nanobody, aSR29, potently neutralizes diverse variants, including XBB.1.5 and BQ.1.1. By fusing aSR29 with the viral receptor ACE2, we created a bispecific molecule that blocks viral entry through two independent mechanisms, achieving a 33-fold increase in neutralization potency. This work provides a practical and scalable strategy for pandemic preparedness, demonstrating that engineered nanobody–ACE2 fusions can serve as effective broad-spectrum therapeutics against emerging SARS-like viruses.

## INTRODUCTION

The SARS-CoV-2 virus (hereafter SARS2) has been circulating for over 5 years, while the 2002 SARS-CoV outbreak (hereafter SARS1) with a higher fatality rate (~11%) remains a haunting memory (1). Previous studies indicate both SARS2 and SARS1 likely originated naturally, with bats as their natural reservoirs. Phylogenetic analyses classify both SARS2 and SARS1 under lineage B of Betacoronavirus (2, 3). To date, at least 73 SARS-like viruses have been identified in animal hosts (4–15). Continuous cross-host transmission enables viral recombination, coupled with mutations and adaptive evolution, increasing the likelihood of novel SARS-like viruses re-emerging to threaten humans (16, 17). Thus, developing broad-spectrum therapeutics against such viruses holds critical strategic importance.

Both SARS1 and SARS2 initiate infection through binding of their spike protein receptor-binding domain (RBD) to ACE2 on host epithelial cells. Although RBD-targeting antibodies exhibit potent neutralizing activity, their efficacy is significantly affected by emerging viral mutations (18–23). For instance, the BQ.1.1 variant carries 20 RBD mutations that render most clinically approved antibodies ineffective or less potent (24–27). Developing antibodies targeting conserved RBD epitopes across SARS-like viruses could counteract viral immune evasion (20).

In addition to conventional antibodies, nanobodies (also known as VHH) have emerged as promising candidates in COVID-19 therapeutics due to their unique structural advantages (28–30). Comprising only the heavy-chain variable region, nanobodies exhibit a molecular weight of merely 13–15 kDa (31–33). Their compact size enables them to recognize epitopes inaccessible to conventional antibodies, and their single-domain architecture facilitates engineering modifications (34). Therefore, the development of broad-spectrum nanobodies targeting both SARS1 and SARS2 holds substantial promise.

Here, we implemented an alternating immunization strategy in alpacas using SARS1 and SARS2 RBDs to elicit broad-spectrum nanobodies targeting conserved epitopes against SARS-like viruses. Four nanobodies, named aSR29, aSR196, aSR347, and aSR348, were finally selected, and their binding affinities were characterized. Moreover, the structure of aSR29/aSR347/SARS1 RBD complex was determined, and the interface analysis reveals that aSR29 binds to a highly conserved epitope on the inner side of the spike. aSR29 robustly neutralizes the pseudotyped viruses of SARS1, SARS2, and Variants of Concern (VOCs). In addition, we engineered a novel aSR29-ACE2-Fc fusion, whose neutralization capacity is significantly enhanced 33-fold against wild-type SARS2 compared to parental aSR29-Fc. This work not only verified an alternating immunization strategy capable of generating broad-spectrum nanobodies but also prompted that the VHH-ACE2 construction can serve as a feasible strategy to effectively combat SARS-like viruses.

## RESULTS

### Four cross-binding nanobodies against SARS1 and SARS2 were successfully screened

**Fig 1:**
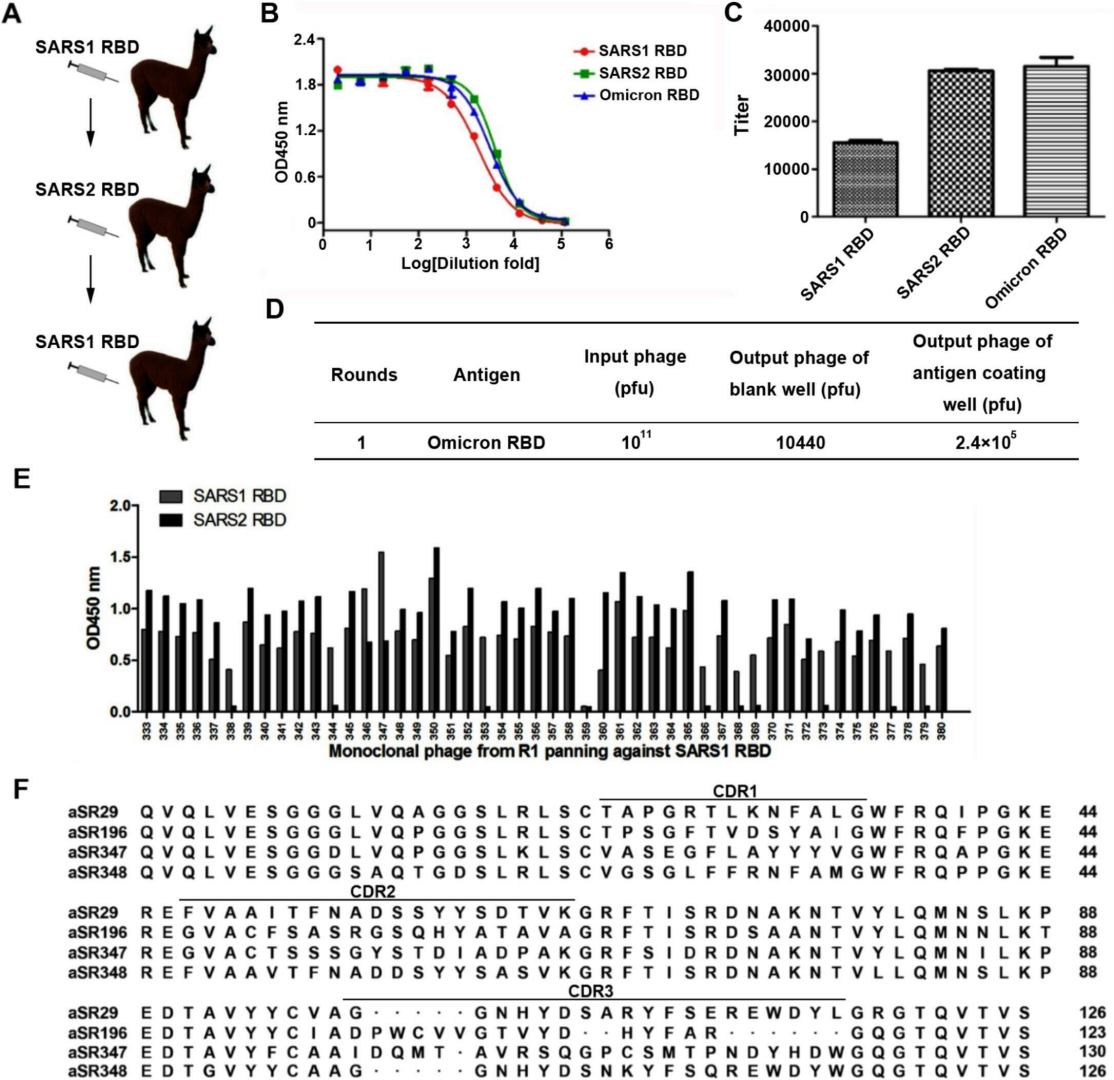
Acquisition of nanobodies targeting SARS1 and SARS2. (**A**) Alternating immunization protocol for alpacas using SARS1 and SARS2 RBD. (**B**) Detection of immunized alpaca serum-binding ability to SARS1, SARS2, and Omicron RBDs by ELISA. Error bars indicate the means ± SD from triplicates. (**C**) Determination of the antibody titer against SARS1, SARS2, and Omicron RBDs in the serum of immunized alpacas by ELISA. Error bars indicate the means ± SD from triplicates. (**D**) Enrichment of phages after panning on Omicron RBD. (**E**) ELISA results for characterizing the binding of the isolated partial nanobodies (Nbs) to SARS1 and SARS2 RBD. (**F**) The amino acid sequence of the isolated aSR29, aSR196, aSR347, and aSR348.

To obtain nanobodies targeting conserved epitopes shared by both SARS1 and SARS2, we immunized an alpaca with three alternating rounds of SARS1 and SARS2 RBD antigens, as shown in Fig. 1A. According to the enzyme-linked immunosorbent assay (ELISA) results, the antibodies in the serum were capable of binding to the RBD of SARS1, SARS2, and the Omicron variant (Fig. 1B). Upon further analysis, the antibody titer against SARS1 RBD exceeds 15,000, and the titers against SARS2 and Omicron RBD are both over 3,000 (Fig. 1C). These results illustrated that the alternating immunization strategy successfully elicited high nanobody production against both SARS1 and SARS2 RBDs in the alpaca.

Next, we successfully constructed a phage-displayed nanobody library. Using the phage display technology, one round of bio-panning was performed with Omicron RBD as the target antigen, yielding an output of 2.4 × 10^5^ phages (Fig. 1D), indicating effective enrichment. Individual phage clones were randomly selected and screened for SARS1/2 RBD-binding activity via phage ELISA (Fig. 1E). Sequencing of positive clones identified four unique nanobody sequences, designated as aSR29, aSR196, aSR347, and aSR348 (Fig. 1F).

Furthermore, we performed surface plasmon resonance (SPR) to verify the four nanobodies’ binding affinity. The results revealed that aSR29, aSR196, aSR347, and aSR348 nanobodies bound SARS2 RBD with high affinities (*K*_D_ values ranging from 0.5 nM to 1.48 pM) and SARS1 RBD with *K*_D_ values between 2.82 nM and 0.034 pM (Table 1; Fig. S1A through H), prompting remarkable cross-binding capacity.

**TABLE 1:** Binding affinity *K*_D_ value (nM) of Nb-Fc proteins for the tested RBDs of SARS1 and SARS2 detected by SPR

Antibody	*K*_D_ (nM)	
	SARS1 RBD	SARS2 RBD
aSR29	0.00481	0.26
aSR196	2.82	0.501
aSR347	0.151	0.00148
aSR348	0.0000338	0.0043

### Characterization of identified Nb binding to VOCs by SPR

We further employed SPR to validate the broad-spectrum binding capabilities of aSR29, aSR196, aSR347, and aSR348 against VOCs. All four nanobodies displayed strong binding affinity toward the RBDs of BA.1, BA.2, BA.5, XBB, and BQ.1.1 and the XBB.1.5 spike, with potencies comparable to that observed for the wild-type SARS2 RBD. The binding affinities of aSR29, aSR196, aSR347, and aSR348 for the above six VOCs ranged between 0.25 and 0.85 nM, 0.11 and 0.59 nM, 0.27 and 78.7 pM, and 0.016 pM and 0.57 nM, respectively (Table 2; Fig. S2A through D). These findings suggest that the nanobodies likely target conserved epitopes on the RBD.

**TABLE 2:** Binding affinity *K*_D_ values (nM) of Nb-Fc proteins for the tested antigens of VOCs detected by SPR

Antigen	*K*_D_ (nM)			
	aSR29	aSR196	aSR347	aSR348
BA.1 RBD	0.414	0.394	0.006	0.0595
BA.2 RBD	0.797	0.393	0.000928	0.2
BA.5 RBD	0.396	0.306	0.00199	0.247
XBB RBD	0.849	0.588	0.0787	0.569
BQ.1.1 RBD	0.252	0.306	0.000303	0.000016
XBB.1.5 spike	0.496	0.107	0.000272	0.39

**Fig 2:**
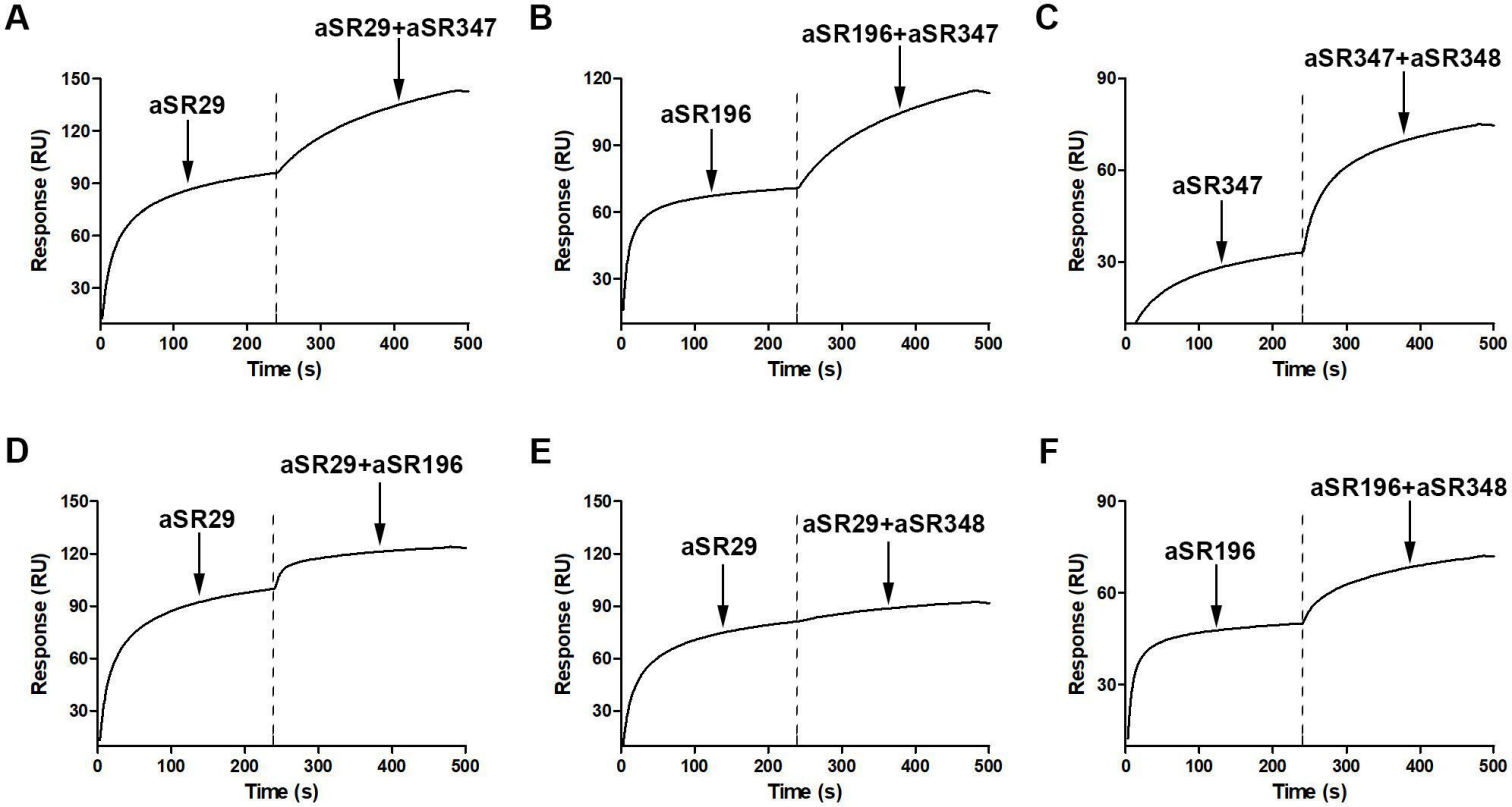
Competitive binding of aSR29, aSR196, aSR347, and aSR348 to SARS2 RBD by SPR. (**A–F**) Competitive binding assays of aSR29 and aSR347, aSR196 and aSR347, aSR347 and aSR348, aSR29 and aSR196, aSR29 and aSR348, and aSR196 and aSR348, respectively.

To map the epitopes recognized by aSR29, aSR196, aSR347, and aSR348, we performed SPR-based competition assays to assess their overlap on SARS2 RBD. After saturating the antigen with one nanobody, we sequentially injected another nanobody to determine potential epitope competition by monitoring the response value changes. The results revealed no competition between aSR347 and the other three nanobodies (Fig. 2A through C), while potential competition was observed among aSR29, aSR196, and aSR348 (Fig. 2D through F). This indicates that aSR29, aSR196, and aSR348 likely recognize adjacent conserved epitopes on the RBD, whereas aSR347 may bind to a more distinct epitope.

### Structure analysis of aSR29 and aSR347 binding to SARS1 RBD

**Fig 3:**
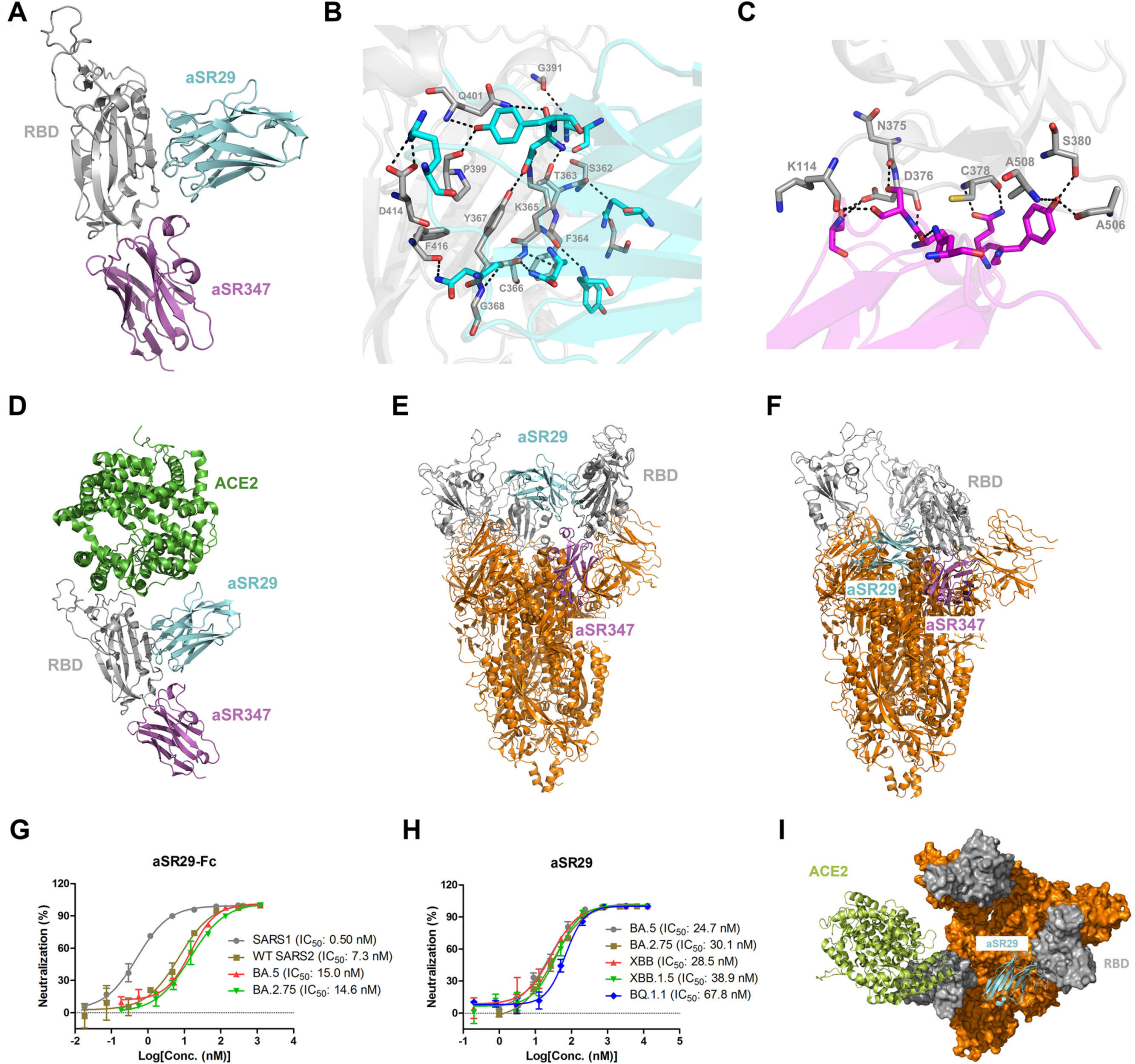
Molecular mechanisms of aSR29-mediated broad-spectrum neutralization against SARS1, SARS2, and their VOCs. (**A**) Overall structure of the aSR29:aSR347:SARS1 RBD complex (Protein Data Bank [PDB] ID: 8WCG). (**B**) Magnified view of the aSR29:SARS1 RBD-binding interface, with interacting residues shown as sticks and dashed lines indicating hydrogen bonds and salt bridges. (**C**) Magnified view of the aSR347:SARS1 RBD-binding interface. (**D**) Structural alignment of the aSR29:aSR347:SARS1 RBD complex with ACE2:SARS2 RBD. (**E and F**) Structural alignment of the aSR29:aSR347:SARS1 RBD complex with open (**E**) and closed (**F**) RBD conformations on the intact SARS2 spike. (**G**) Neutralization activity of aSR29-Fc against SARS1, ancestral SARS2, BA.5, and BA.2.75 pseudoviruses. (**H**) Neutralization activity of aSR29 against BA.5, BA.2.75, XBB, XBB.1.5, and BQ.1.1 pseudoviruses. (**I**) The epitope recognized by aSR29 is located at the bottom inner-facing region of the RBD.

To further elucidate the binding mechanisms of nanobodies with SARS1 and SARS2 RBDs, we screened crystallization conditions for the ternary complexes of SARS1 or SARS2 RBD with aSR29:aSR347, aSR196:aSR347, and aSR347:aSR348. Ultimately, we obtained high-quality single crystals of the aSR29:aSR347:SARS1 RBD complex under 15% (wt/vol) PEG 6000 and 0.1 M TRIS at pH 7.5. X-ray diffraction data were collected, and the complex structure was successfully resolved at 2.68 Å resolution using molecular replacement (Fig. 3A). Data collection and model refinement statistics of the structure are shown in Table S1. Analysis of the antibody-antigen binding interface revealed that aSR29 interacts with twelve SARS1 RBD residues (S362, T363, F364, K365, C366, Y367, G368, G391, P399, Q401, D414, and F416) (Fig. 3B), whereas aSR347 binds to seven residues (K114, N375, D376, C378, S380, A506, and A508) (Fig. 3C).

Structure alignment of the aSR29:aSR347:SARS1 RBD with the ACE2: SARS2 RBD complex demonstrated that neither aSR29 nor aSR347 competes with ACE2 for binding to SARS2 RBD (Fig. 3D). Superimposition of the aSR29:aSR347:SARS1 RBD onto the full-length spike trimer revealed that aSR29 binds to an RBD core region on the inner side of RBD. Furthermore, due to steric hindrance, aSR29 binds specifically to the open spike conformation but not to the closed spike. Besides, since aSR347 binds to the bottom of the RBD, it results in its inability to bind to either the open or closed spike conformation (Fig. 3E and F).

### aSR29 efficiently neutralizes VOCs’ pseudovirus

Since SPR results showed that aSR29 has a higher ka value for binding to the RBD compared to aSR347 (Fig. S1) and structural information indicated that aSR347 cannot effectively bind to the RBD on the intact spike, we selected aSR29 for further investigation. We first tested the broad-spectrum neutralizing activity of aSR29. Pseudovirus neutralization assays demonstrated that aSR29-Fc neutralized SARS1, SARS2, as well as BA.5 and BA.2.75 with IC_50_ values of 0.5, 7.3, 15, and 14.6 nM, respectively (Fig. 3G). Moreover, aSR29 maintained high-potency neutralizing activity against XBB.1.5 and BQ.1.1, with IC_50_ values of 38.9 and 67.8 nM, respectively (Fig. 3H).

These results are consistent with structural insights. The SARS1 amino acid residues bound by aSR29 correspond to S375, T376, F377, K378, C379, Y380, G381, G404, P412, Q414, D427, and F429 on SARS2. Among these, only S375 and T376 are mutated in BA.2, BA.5, XBB, and BQ.1.1, while the other ten residues contacted by aSR29 remain unmutated in most recent variants such as JN.1, KP.3, XEC, and XFG (Fig. S4). Notably, S375 and T376 (correspond to S362 and T363 on SARS1 RBD) are positioned at the periphery of the aSR29-RBD binding interface (Fig. S5), suggesting their mutations may minimally affect binding.

These findings confirm that aSR29 targets a highly conserved epitope. Further structural analysis revealed that this epitope is located at the base of the RBD, oriented toward the inner side of the spike (Fig. 3I). Steric hindrance likely prevents conventional antibodies from accessing this region, thereby explaining the lack of viral escape mutations at this site despite vaccine-induced immunity.

### Engineered aSR29-ACE2-Fc fusion enhances SARS2 neutralization

**Fig 4:**
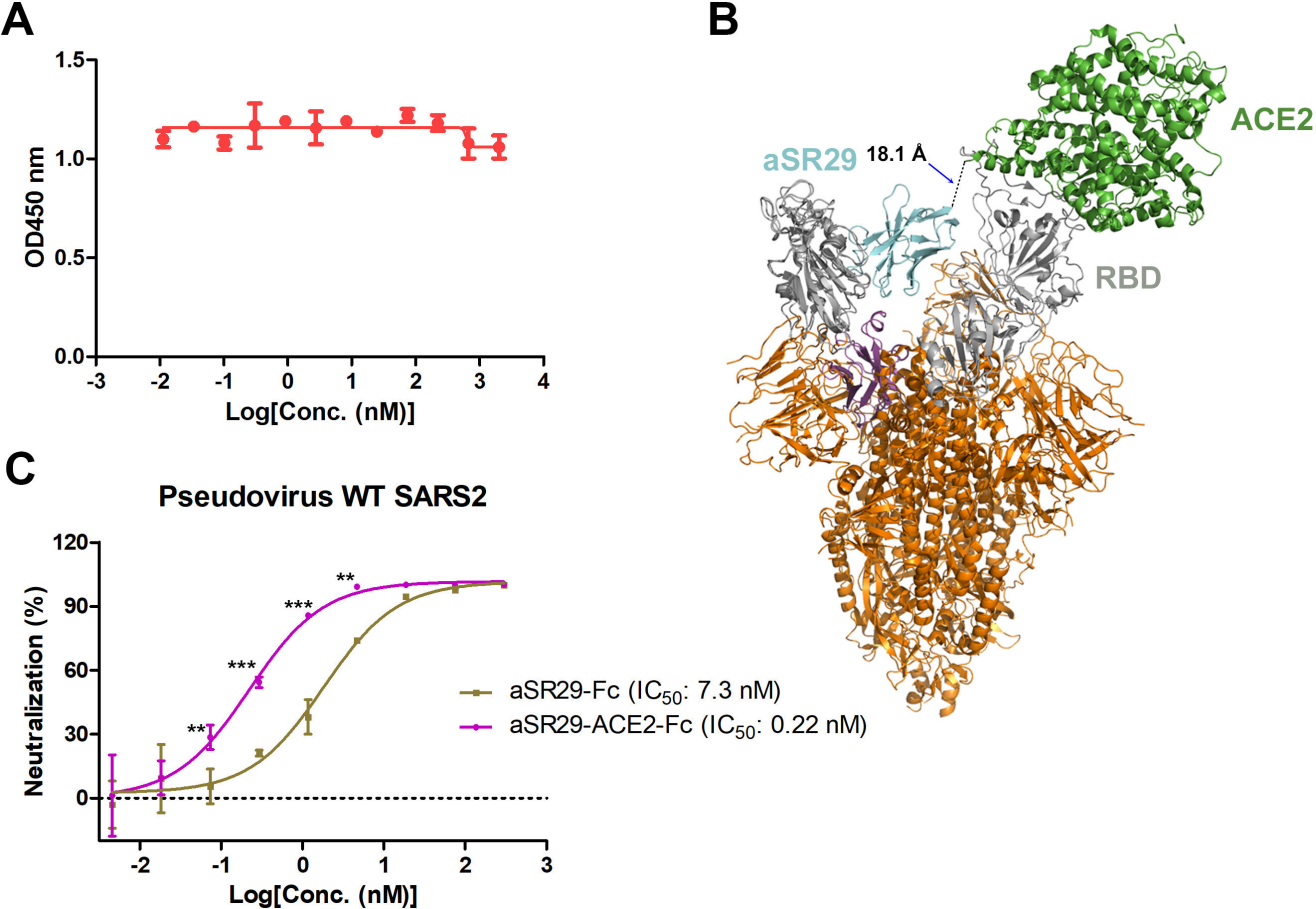
Design and characterization of aSR29-ACE2-Fc. (**A**) The competition ability between aSR29 and ACE2-Fc for binding to SARS2 RBD was performed by competitive ELISA. (**B**) The structure of the aSR29:RBD complex was aligned with the full SARS2 spike, and the distance from the C-terminus of aSR29 to the N-terminus of the indicated ACE2 was measured. (**C**) The neutralizing activity of aSR29-ACE2-Fc against prototypic SARS2 pseudovirus. The *P* values of the two experimental groups were statistically analyzed using two-way ANOVA in Prism software. ** indicates *P* < 0.01, highly significant; *** indicates *P* < 0.001, extremely significant.

Based on the structural analysis (Fig. 3D), ACE2 and aSR29 bind to non-overlapping epitopes on the RBD without steric hindrance. Competitive ELISA further confirmed this result (Fig. 4A). We hypothesized that an aSR29-ACE2 fusion protein, engineered to bind the spike protein simultaneously by engaging two distinct RBDs, would not only enhance the overall binding affinity but also block the spike’s interaction with host cell-surface ACE2, leading to improved viral neutralization. Structural analysis reveals that the C-terminus of aSR29 and the N-terminus of ACE2 are separated by 18.1 Å when the two molecules are bound to distinct RBDs on the spike protein (Fig. 4B). To eliminate structural stretching stress, ensure proper antibody folding and free orientation, and avoid the drawbacks of long linkers such as tangling and protease susceptibility (35), we connected the C-terminus of aSR29 to the N-terminus of ACE2 with a 36 Å flexible linker (two GGGGS repeats), which was then fused to a human IgG1 Fc domain to generate the bispecific fusion protein aSR29-ACE2-Fc.

As anticipated, aSR29-ACE2-Fc exhibited a ~ 33 fold improvement in neutralizing activity against SARS2 compared to aSR29-Fc, achieving an IC_50_ of 0.22 nM (Fig. 3G and 4C). Together, these data demonstrate the successful construction of a bispecific agent and underscore its promise as a broad-spectrum neutralizer against SARS1, SARS2, and related VOCs.

## DISCUSSION

Pathogenic microorganisms exhibit high genetic diversity and can render host immunity, vaccines, and treatments ineffective through genetic mutations. As mutations accumulate, the prevalence of variants such as Omicron subvariants (e.g., XBB and BQ.1.1) poses severe challenges to developing highly potent, durable, and broad-spectrum anti-SARS2 antibodies and even necessitates the consideration for future potential sarbecovirus threats. Consequently, an increasing number of methods are being developed to counter viral immune evasion. There are 58 surface-exposed residues showing conservation across major SARS2 variants (Alpha, Beta, Gamma, Delta, Lambda, Kappa, and Omicron). Notably, 16 of Omicron’s 17 RBD mutations (excluding S375F) occurred outside these conserved sites (Fig. S3). This demonstrates the feasibility of developing antibodies targeting these conserved epitopes with potential pan-SARS-like virus activity and enhanced mutational resistance.

Liu et al*.* demonstrated that adding a novel STING agonist adjuvant, CF501, to the recombinant protein SARS2 RBD-Fc vaccine effectively enhanced neutralizing antibody titers against SARS2 VOCs in the sera of rhesus macaques (36). Li et al. introduced the P1263L mutation into the COVID-19 VLP mRNA vaccine, which was shown to enhance antigen presentation on VLPs and elicit higher neutralizing antibody titers in mice (37). Whether through adjuvant addition or introducing mutation, both approaches address the persistent challenge of viral mutation. In the design of vaccines from the perspective of achieving broad-spectrum protection, one may also choose to immunize with vaccines containing conserved antigens from multiple coronaviruses. Given the sequence differences among antigens from different coronaviruses, mixing these proteins can increase the probability that the immune system will generate or enhance immune responses against conserved epitopes. Consequently, this leads to the generation of corresponding memory cells, the production of broad-spectrum antibodies, and the realization of broad-spectrum protective effects (38). In this research, we used an alternating SARS1/SARS2 RBD immunization strategy, which successfully obtained nanobodies targeting SARS1/SARS2 RBD and VOCs in alpaca. By alternating antigen components, host immune tolerance can be mitigated and immune responses enhanced. Thus, this approach represents an effective strategy for rapidly eliciting broad-spectrum, cross-reactive nanobodies from immunized animals to counter emerging viral outbreaks.

More than 40 nanobodies against the SARS2 RBD have been reported worldwide (39–63). Analysis of binding interfaces indicates that aSR29 targets an epitope similar to those of class II(A) nanobodies (e.g., VHH72, 1–8, 1–25, 2–31, 2–34, and 2–57) (63), which bind a hydrophobic core (residues 377–386). Notably, aSR29 additionally engages residues P412, Q414, D427, and F429, characteristic of class II(B) antibodies. This extended interface enhances its neutralizing breadth and potency against mutant strains (Fig. 3G and H), making it a promising candidate for combating future SARS-like virus outbreaks.

Before the virus invades host cells, the spike protein exhibits reversible “breathing” motions. After the RBD binds to ACE2, it drives the gradual outward expansion of the S1 subunits, inducing a more open conformation of the spike RBD (64, 65). We engineered the aSR29-ACE2 fusion with the aim of achieving a synergistic effect: while ACE2 binds to the RBD to prevent the virus from attaching to host cells, and its high-affinity binding locks the spike in an open conformation, that is more favorable for the nanobody to bind to the inner side of the RBD. Researchers converted a fusion antibody into one with broad-spectrum neutralizing activity by linking ACE2 to the N-terminus of an scFv derived from a non-neutralizing, cross-reactive antibody (66), and other researchers have also linked ACE2 to the C-terminus of the heavy chain of conventional antibodies to enhance their ability to neutralize the SARS2 (67). Our study is distinct in utilizing a nanobody platform to conjugate with ACE2. The small size and structural flexibility of nanobodies offer significant advantages for protein engineering and function improvement. Consistently, our engineered aSR29-ACE2-Fc exhibited a 33-fold enhancement in neutralizing SARS2 pseudovirus compared to the parental antibody (Fig. 3E through I). Therefore, the VHH-ACE2 design represents a promising strategy to enhance both the affinity of the fusion protein and its resilience against viral immune evasion.

Some studies have demonstrated the use of the ACE2 α1 peptide for viral neutralization; however, its application is limited by poor stability and reduced neutralizing potency against VOCs (68, 69). Building on the findings in this study, leveraging the conserved residues at the ACE2-RBD binding interface for the artificial intelligence-assisted *de novo* design of ACE2-mimetic proteins offers a promising route toward achieving broad-spectrum sarbecovirus neutralization. Rationally engineering these proteins in fusion with aSR29 may represent an effective strategy to counter the potential future outbreaks of sarbecoviruses.

## MATERIALS AND METHODS

### Protein preparation

SARS1 RBD (amino acids 320 to 516, NCBI accession no. NC_004718) and SARS2 RBD (amino acids 321 to 591, NCBI accession no. MN908947) proteins were prepared as in our previous study (29). Briefly, the coding sequences were cloned into the pTT5 vector, with the C terminal containing a TEV cleavage site and a human IgG1 Fc. All recombinant vectors were constructed using the Gibson assembly method (30). The recombinant vectors were transiently transfected into HEK293F cells with polyethyleneimine (Polyscience). After 3 days of expression, the fusion protein was purified from the cell supernatant using a protein A column (GE Healthcare). After digestion with TEV protease, the Fc fragment was removed by a second protein A column purification, and the TEV protease was removed by a nickel column (GE Healthcare). VHH, VHH-Fc, and VHH-ACE2-Fc, including aSR29, aSR129, aSR347, aSR348, aSR29-Fc, aSR129-Fc, aSR347-Fc, aSR348-Fc, and aSR29-ACE2-Fc, were also prepared similarly. Omicron BA.1, BA.2, BA.5, XBB, and BQ.1.1 RBD proteins were purchased from Sino Biological.

### Alpaca immunization strategy

A female alpaca was immunized twice by subcutaneous injection and once by intramuscular injection, each with 500 μg of the antigen in phosphate-buffered saline (PBS), emulsified with an equal volume of Freund’s adjuvant (Sigma-Aldrich). The alpaca received the primary immunization with SARS2 RBD, followed by a secondary immunization with SARS1 RBD 2 weeks later, and a third immunization with a combination of SARS1 RBD and SARS2 RBD after another 2 weeks.

### Detection of alpaca serum antibody titers

Venous blood was collected 2 weeks after the third immunization. Serum was separated by centrifugation. Immuno MaxiSorb plates were coated with SARS1, SARS2, or Omicron RBD. Four times serially diluted serum was added to the RBD-coated wells and incubated for 1 h. After washing four times with PBST, bound alpaca-derived antibodies were detected with horseradish peroxidase (HRP)-conjugated anti-alpaca antibody (Sino Biological). After incubation for 1 h at room temperature (RT), the plates were washed, 100 μL per well of TMB was added and incubated in the dark for 5 min, and 50 μL per well of 1 M sulfuric acid was added to stop the reaction. The OD450 was read by a Synergy H1 plate reader (Biotek). The data were analyzed using GraphPad Prism software.

### Construction and screening of phage libraries

#### Construction of phage libraries

More than 1 × 10⁷ lymphocytes were isolated from peripheral blood using a density gradient separation medium (density: 1.077 g/mL; Sigma). Total RNA was extracted with a commercial kit (TAKARA) and reverse-transcribed into cDNA. The VHH-encoding gene fragments were then amplified with alpaca VHH-specific primers, cloned into the phagemid vector pR2 via Gibson Assembly, and transformed into TG1 competent cells to generate the phage-display library (30).

#### Phage display

Immuno MaxiSorb plates (Nunc) were coated with 0.1 mL of Omicron RBD (100 μg/mL) and incubated overnight at 4°C. Control wells without antigen coating were included in each round of panning. After blocking with MPBS (PBS containing 5% skim milk) for 2 h at room temperature, 1 × 10¹¹ PFU of library phage particles were added for the first round of selection. The wells were washed 30 times with PBST (PBS containing 0.1% Tween 20) to remove unbound phages. Bound phages were eluted by incubating with 100 μL of trypsin (0.5 mg/mL) for 1 h at room temperature. The eluted phages were then used to infect *Escherichia coli* TG1 cells for titer determination and subsequent amplification.

A total of 475 individual clones obtained from one round of panning were subjected to monoclonal phage ELISA screening. Briefly, each monoclonal phage was rescued using helper phage KM13 and then added to wells coated with SARS1 RBD (2 μg/mL). After a 1-h incubation at room temperature, the wells were washed four times with PBST. Subsequently, horseradish peroxidase (HRP)-conjugated anti-M13 antibody (Sino Biological) was added. Following another four washes with PBST, TMB substrate (Beyotime) was added to each well and incubated in the dark at room temperature for 5 min. The chromogenic reaction was terminated by adding 50 μL of 1 M sulfuric acid, and the absorbance at 450 nm (OD₄₅₀) was measured using a Synergy H1 plate reader (BioTek).

Clones exhibiting OD₄₅₀ values above 0.5 were considered positive and were further analyzed for their binding capacity to SARS2 RBD using the same ELISA procedure. Data were analyzed with GraphPad Prism software. After verification through this two-step monoclonal phage ELISA, the positive clones were selected for sequencing (General Biolotech).

### Competitive ELISA

To assess the competitive binding between aSR29 and ACE2 for SARS2 RBD, a competitive ELISA was performed. Immuno MaxiSorb plates were coated with SARS2 RBD. Serial dilutions of aSR29 were prepared in 100 nM ACE2-Fc solution (1:4 dilution series) and added to the RBD-coated wells, followed by incubation for 1 h at room temperature. After washing four times with PBST, bound ACE2-Fc was detected using an HRP-conjugated anti-human IgG1 Fc antibody (Sino Biological). Following another 1-h incubation at room temperature and subsequent washing, 100 μL of TMB substrate was added per well and incubated in the dark for 5 min. The reaction was stopped with 50 μL of 1 M sulfuric acid per well, and the absorbance at 450 nm (OD₄₅₀) was measured using a Synergy H1 plate reader (BioTek). Data were analyzed with GraphPad Prism software.

### SPR

SPR measurements were conducted at 25°C using a Biacore 8K system (Cytiva). The RBD protein was diluted to 10 μg/mL in sodium acetate buffer (pH 4.5) and immobilized on an activated CM5 sensor chip (Cytiva). All samples were exchanged into running buffer (PBS containing 0.05% Tween 20, pH 7.4) and injected at a flow rate of 5 μL/min. A reference flow cell without the immobilized protein was used for background subtraction.

For affinity measurements, a series of concentrations of each nanobody were injected over the sensor surface. The chip was regenerated between cycles by injection of 50 mM NaOH for 60–120 s. Sensorgrams were analyzed using the Biacore Evaluation Software, and binding kinetics were fitted to a 1:1 Langmuir binding model.

To assess epitope competition between aSR29, aSR196, aSR347, and aSR348, a sequential injection assay was performed. First, one nanobody (200 nM) was injected for 120 s to achieve binding saturation. Without regenerating the chip, a mixture containing an equimolar concentration (200 nM each) of the first and a second nanobody was then injected for 120 s. An increase in response units during the second injection indicated that the two nanobodies bind to non-overlapping epitopes and do not compete for RBD binding.

### Crystallization and data collection

The complex was formed by incubating purified SARS1 RBD with aSR29 and aSR347 at a molar ratio of 1:1.2:1.2. Excess nanobodies were removed by gel filtration chromatography, and the protein complex was concentrated to 10 mg/mL for crystallization trials. Crystals were grown at 18°C using the sitting-drop vapor-diffusion method by mixing 0.2 µL of the protein complex with an equal volume of the reservoir solution. The crystallization condition contained 0.1 M Tris-Cl (pH 7.5) and 15% (wt/vol) PEG 6000. For cryo-protection, crystals were briefly soaked in the reservoir solution supplemented with 25% (vol/vol) ethylene glycol and then flash-cooled in liquid nitrogen. X-ray diffraction data were collected at the BL10U2 beamline of the Shanghai Synchrotron Radiation Facility (SSRF) using a wavelength of 0.97902 Å.

### Structural determination

Data were processed with XDS (70). Initial phases were solved by the molecular replacement method with Phaser (71) from the CCP4i program package (72) using SARS1 RBD (PDB ID: 7LM9), VHH 2-31 (PDB ID: 8CWV), and VHH E (PDB ID: 7KN5) as search models for the SARS1 RBD: aSR29: aSR347 complex. Subsequent model building and refinement were achieved using COOT and Phenix (25). The structural data of SARS1 RBD: aSR29: aSR347 complex have been deposited in PDB under accession codes 8WCG. All structural figures were prepared with PyMOL.

### Pseudovirus neutralization assay

Pseudovirus neutralization assays were performed to evaluate the neutralizing activities of aSR29-Fc, aSR29, and aSR29-ACE2-Fc against SARS1, wild-type (WT) SARS2, and the Omicron subvariants BA.5, BA.2.75, XBB, XBB.1.5, and BQ.1.1. HIV-1-based pseudoviruses carrying the SARS1 spike protein and a luciferase reporter gene were prepared as previously described (21). Pseudoviruses for the other variants were obtained commercially (Vazyme, China).

The assay was conducted as previously reported (21). Briefly, ACE2-293T cells were seeded in 96-well plates at 2.5 × 10⁴ cells per well and cultured overnight. Threefold serial dilutions of antibodies in DMEM supplemented with 10% FBS were incubated with an equal volume of pseudovirus at 37°C for 1 h. The antibody-pseudovirus mixtures were then added to the ACE2-293T cell monolayer. After 48 h of incubation, cells were lysed, and luciferase activity was measured using Bright-Lite detection reagent (Vazyme, DD1204) on a microplate luminescence reader (TECAN Spark 10M). Wells containing cells only (no virus, no antibody) served as blank controls, and wells with virus but no antibody were used as positive (virus) controls.

Neutralization percentage was calculated as follows:


$$\text{Neutralization (\textbackslash{}\%)}=\left[1-\frac{\text{sample RLU}-\text{blank RLU}}{\text{virus control RLU}-\text{blank RLU}}\right]\times 100\%.$$

## Data Availability

The atomic coordinates of the SARS1 RBD/aSR29/aSR347 ternary complex are publicly available in the Protein Data Bank (PDB) under accession code 8WCG. The data that support the findings of this study are openly available in this article and are also available from the corresponding author upon request.

## SUPPLEMENTAL MATERIAL

Figure S1 jvi.00608-26-s0001.tif Characterization of binding affinity of four isolated Nbs using SPR.

Figure S2 jvi.00608-26-s0002.tif Characterization of binding affinity of four isolated Nbs to VOCs using SPR.

Figure S3 jvi.00608-26-s0003.tif Distribution of fully conserved SARS-like virus amino acids on the SARS2 RBD.

Figure S4 jvi.00608-26-s0004.tif Sequence alignment of RBDs from SARS1, WT SARS2, and SARS2 mutant strains.

Figure S5 jvi.00608-26-s0005.tif Display of mutated residues and the aSR29 binding site on the SARS2 RBD mutants.

Supplemental material jvi.00608-26-s0006.docx Table S1; legends for Fig. S1 to S5.

ASM does not own the copyrights to Supplemental Material that may be linked to, or accessed through, an article. The authors have granted ASM a non-exclusive, world-wide license to publish the Supplemental Material files. Please contact the corresponding author directly for reuse.
