# Supplementary material for: ACE2-fused nanobody targeting a cryptic RBD epitope broadly neutralizes SARS-like viruses
Source: J Virol. 2026 Jul 30;100(8):e00608-26. doi: 10.1128/jvi.00608-26 (PMC13483450; doi:10.1128/jvi.00608-26)
Supplement: Supplementary file 1 [file jvi.00608-26-s0006.docx]

**Table S1.** Data collection and refinement statistics

|  | SARS1 RBD (320-516)/ aSR29/ aSR347 |
| --- | --- |
| Data collection | BL10U2 |
| Wavelength(Å) | 0.97902 |
| Space group | *P2_1_ 2_1_ 2_1_* |
| Unit cell parameters |  |
| a, b, c(Å) | a=76.852,  b=134.679,  c=136.688 |
| α(°)  β(°)  γ(°) | α=90  β=90  γ=90 |
| Resolution range(Å) | 95.93-2.6 (2.74-2.6) |
| Monomers in an asymmetric unit | 2 |
| Unique reflections | 44083 (6390) |
| Average redundancy | 7.7(7.97) |
| Completeness (%) | 99.1(99.7) |
| R_merge_ (%)^a^ | 12.1(119.1) |
| I/σ(I) | 15.1(2.9) |
| Refinement Statistics |  |
| Resolution range(Å) | 47.967-2.599 |
| R_factor_ (%)^b^ | 23.2 |
| R_free_ (%)^c^ | 27.9 |
| RMSD bond lengths(Å) | 0.003 |
| RMSD bond angles(°) | 0.6 |
| Mean B factors(Å^2^) |  |
| Protein | 57.06 |
| Ligand | 85.202 |
| water | 51.834 |
| No. of non-hydrogen protein atoms | 6983 |
| No. of ligand atoms | 30 |
| No. of water oxygen atoms | 98 |
| Ramachandran plot^d^ |  |
| Favored (%) | 94.94 |
| Outliers (%) | 0.11 |
| PDB entry | 8WCG |

^a^$R\text{merge}={\sum_{hkl} \sum_{i} \left| I_{i}\left( \left. hkl \right) \right.-\left\langle I\left( \left. \left. hkl \right) \right\rangle\right. \right. \right|}/{\sum_{hkl} \sum_{i} I_{i}\left( \left. hkl \right) \right.}$, where *I_i_(hkl)* is the intensity of *ith* observation and <I(hkl)> is the mean value for reflection hkl.

^b^$R\text{work}\text{=}{\sum_{hkl} \left| \left| F_{obs}\left| - \right.\left| F_{cacl} \right. \right| \right|}/{\sum_{hkl} \left| F_{obs} \right|}$, where F_obs_ and F_calc_ are the observed and calculated structure-factor amplitudes, respectively.

^c^R_free_ is calculated same as R_work_ with 5% reflections, which were selected randomly from the refinement process.

^d^The categories were defined by PROCHECK.

**Supplementary Figure legend**

**Figure S1.** Characterization of binding affinity of four isolated Nbs using SPR. The SARS2 RBD binding kinetics of aSR29 (**A**), aSR196 (**B**), aSR347 (**C**), and aSR348 (**D**) were measured by SPR, respectively. The SARS1 RBD binding kinetics of aSR29 (**E**), aSR196 (**F**), aSR347 (**G**), and aSR348 (**H**) were measured by SPR, respectively. The SARS1 or SARS2 RBD was immobilized onto a CM5 sensor chip. The Nbs with serial 1:1 dilutions were injected and monitored by using the Biacore 8K system. The actual responses (colored lines) and the data fitted to a 1:1 binding model (black dotted lines) are shown. *K*_D_, equilibrium dissociation constant; *k*_a_, association constant; *k*_d_, dissociation constant.

**Figure S2.** Characterization of binding affinity of four isolated Nbs to VOCs using SPR. The binding kinetics of aSR29 (**A**), aSR196 (**B**), aSR347 (**C**), and aSR347 (**D**) to BA.1, BA.2, BA.5, XBB, BQ.1.1 RBD, and XBB.1.5 spike were measured by SPR. The antigen proteins were immobilized onto a CM5 sensor chip. The Nbs with serial 1:1 dilutions were injected and monitored by using the Biacore 8K system. The actual responses (colored lines) and the data fitted to a 1:1 binding model (black dotted lines) are shown. *K*_D_, equilibrium dissociation constant; *k*_a_, association constant; *k*_d_, dissociation constant.

**Figure S3.** Distribution of fully conserved SARS-like virus amino acids on the SARS2 RBD. Conserved residues exposed on the surface are shown in red, while those buried or inaccessible are shown in green. Mutation sites from representative1 variants—including Alpha, Beta, Gamma, Delta, Lambda, Kappa, and Omicron—are highlighted in purple. Glycosylation sites are indicated in blue. The figure was generated based on the PDB: 7KMS structure and rendered in surface representation.

**Figure S4.** Sequence alignment of RBDs from SARS1, WT SARS2, BA.5, BA.2.75, XBB.1.5, BQ.1.1, JN.1, KP.3, XEC, LP.8.1, XFG, NB.1.8.1, XFG.3, and LF.7. Mutated residues are highlighted with distinct background colors. Residues that bind to aSR29 are shown in red.

**Figure S5.** Display of mutated residues and the aSR29 binding site on the SARS2 RBD mutants. Mutation sites are summarized from WT SARS2, BA.5, BA.2.75, XBB.1.5, BQ.1.1, JN.1, KP.3, XEC, LP.8.1, XFG, NB.1.8.1, XFG.3, and LF.7, and are marked in red. aSR29 is shown in cyan. RBD residues that bind to aSR29 are shown in limon; residues S375F and T376A are also highlighted in purple.
